# Transactivation of Epidermal Growth Factor Receptor by G Protein-Coupled Receptors: Recent Progress, Challenges and Future Research

**DOI:** 10.3390/ijms17010095

**Published:** 2016-01-12

**Authors:** Zhixiang Wang

**Affiliations:** The Department of Medical Genetics and Signal Transduction Research Group, Faculty of Medicine and Dentistry, University of Alberta, Edmonton, AB T6G 2H7, Canada; zhixiang.wang@ualberta.ca; Tel.: +1-780-492-0710

**Keywords:** EGF receptor, G protein-coupled receptors, mechanisms, cancer

## Abstract

Both G protein-coupled receptors (GPCRs) and receptor-tyrosine kinases (RTKs) regulate large signaling networks, control multiple cell functions and are implicated in many diseases including various cancers. Both of them are also the top therapeutic targets for disease treatment. The discovery of the cross-talk between GPCRs and RTKs connects these two vast signaling networks and complicates the already complicated signaling networks that regulate cell signaling and function. In this review, we focus on the transactivation of epidermal growth factor receptor (EGFR), a subfamily of RTKs, by GPCRs. Since the first report of EGFR transactivation by GPCR, significant progress has been made including the elucidation of the mechanisms underlying the transactivation. Here, we first provide a basic picture for GPCR, EGFR and EGFR transactivation by GPCR. We then discuss the progress made in the last five years and finally provided our view of the future challenge and future researches needed to overcome these challenges.

## 1. Introduction

G protein-coupled receptors (GPCRs) and receptor-tyrosine kinases (RTKs) are the two largest groups of membrane receptors. They transduce various extracellular stimulus into intracellular signals to regulate almost all kinds of cellular functions. While it was traditionally believed that the main function of GPCR is to catalyze the production of second messages to regulate cell metabolism and the main function of RTKs is to regulate cell growth and differentiation, recent evidence indicates that both of them activate a large intracellular signaling network that controls multiple cell functions [1,2,3,4]. The discovery of the cross-talk between GPCR and RTKs now integrates these two signaling networks together [5,6]. Many RTKs including epidermal growth factor (EGF) receptor (EGFR), platelet-derived growth factor receptor (PDGFR), and Trks are transactivated by GPCR, however, EGFR transactivation by GPCR is mostly studied. In this review, we will focus on EGFR transactivation. A few reviews have been published recently to discuss the various aspects of RTK transactivation by GPCR [6,7,8]. Here we will focus on the progress during last five years and discuss the challenges and future research of this interesting and important research field.

## 2. G Protein-Coupled Receptors

G protein coupled receptors (GPCRs) constitute a large family of plasma membrane receptors, which is involved in diverse intracellular signaling pathways and regulate multiple functions including vasorelaxation, heart rate, sight, olfaction and biorhythms [9]. The human genome encodes approximately 800 GPCRs, which are grouped into three main classes (A–C) based on sequence homology [2,10]. Recently, GPCRs are also classified into six classes, Rhodopsin-like, Secretin receptor family, Metabotropic glutamate/pheromone, Fungal mating pheromone receptors, Cyclic AMP receptors, and Frizzled/Smoothened [2,11]. The class A/Rhodopsin family comprises the largest number of GPCRs with hundreds of receptors. The class A receptors are further divided into several subtypes including serotonin 5HT1B and 5-HT2B receptors, muscarinic acetylcoline M2 and M3 receptors, and four subtype of opioid receptors [2]. The class B/Secretin family GPCR comprise fifteen receptors in human that are activated by peptide endocrine hormones, peptide paracrine factors and neuropeptides [12]. The class C/Glutamate family GPCR comprise fifteen receptors activated by amino acids and ions [11].

GPCRs are integral membrane proteins with seven transmembrane helices. The extracellular loops of the receptor possess two highly conserved cysteine residues. These two cysteine residues form disulfide bonds, which stabilizes the structure of the receptor. GPCRs lack intrinsic enzymatic activity and function through the coupled heterotrimeric G proteins. The heterotrimeric G protein contains three subunits: Gα, Gβ, and Gγ. Ligand binding to the GPCRs stimulates the dissociation of G proteins into GTP-bound Gα, and Gβγ subunits. The disassociated G protein subunits control the activity of many enzymes including kinases, phospholipase C, and adenylate cyclase to generate second messengers. These intracellular second messengers regulate various cell functions. In addition, the G protein-dependent signaling is further complicated by the existence of many G protein subunits, which can stimulate diverse signaling cascades. Thus far, there are 20 Gα, 6 Gβ, and 11 Gγ subunits reported [6].

There are currently only two protein families shown to regulate GPCR activities: GPCR kinases (GRKs) and β-arrestins [1,6]. GRKs controls GPCR activity by phosphorylating their intracellular domains following the release of the coupled G proteins, which allows the binding of β-arrestins to GPCRs to prevent their re-association with the G proteins. It has been shown recently that β-arrestins also function as scaffolds and activator for many signaling proteins. The interaction between GPCR and β-arrestins allows GPCR to activate downstream signaling cascades, independent of G proteins.

## 3. Epidermal Growth Factor Receptor (EGFR)

There are more than 90 known protein tyrosine kinase genes in the human genome; 58 encode transmembrane RTKs distributed into 20 subfamilies including EGFR family [13]. EGFR family receptors consist of four members including EGFR/ErbB1/Her1, ErbB2/Her2, ErbB3/Her3, and ErbB4/Her4. Among EGFR family, ErbB3 has impaired kinase activity and ErbB2 is an orphan receptor. Therefore, ligand-induced heterodimerization is an important mechanism to activate all ErbB receptors [4,14,15]. EGFR is a 170 kD transmembrane protein of a single polypeptide chain. The extracellular domain of EGFR contains 622-amino acids and is heavily glycosylated. It also comprises two cysteine rich regions for ligand binding. The transmembrane domain is a short α-helical peptide with 23 amino acids. The intracellular domain with 542 amino acids comprise a conserved protein tyrosine kinase domain and a C-terminal domain containing the regulatory tyrosine residues [16].

EGFR plays important roles in the regulation of cell growth and development [4,14]. Binding of EGF to EGFR stimulates the dimerization of EGFR and the activation of its kinase, which leads to the trans-autophosphorylation of EGFR [3]. Activated EGFR stimulates various signaling cascades that regulate multiple cell functions including cell proliferation, survival, cytoskeleton reorganization, and motility [4,14]. EGFR is also rapidly internalized following its binding to EGF [17]. The signaling proteins forming complexes with EGFR include Shc, Grb2, Src, PLC-γ1, Cbl, and the p85α subunit of phosphoinositide 3-kinase (PI3K) [18,19,20]. Many signaling pathways are activated by EGFR. For example, activated EGFR binds to Shc/Grb2, which recruits Sos to the plasma membrane to activate Ras. Ras activation leads to the activation of Raf and mitogen-activated protein kinase (MEK). Extracellular signal-regulated kinases (ERKs), activated by MEK, activate transcription factors such as Elk-1 directly or activate c-fos and SRF through RSK [20,21,22,23]. The activation of PLC-γ1 by EGFR regulates cell mitogenesis and migration [24,25,26,27]. The activated EGFR also stimulates the activation of PI3K-AKT pathway, which protects the cell from undergoing apoptosis [28,29,30].

## 4. Transactivation of EGFR by GPCRs

Ullrich’s group provide the first evidence supporting the transactivation of GPCRs by RTK. They showed that EGFR and ErbB2 were rapidly phosphorylated following the addition of various GPCR agonists including lysophosphatidic acid, thrombin, and endothelin-1 in Rat-1 cells [31]. The activation of EGFR and ErbB2 was blocked when EGFR kinase was inhibited either by the inhibitor AG1478 or by the expression of dominant-negative EGFR [31]. They further showed that EGFR transactivation occurred in diverse cell types and different types of G proteins [32]. Afterwards, transactivation of EGFR by GPCR has been reported for various receptor tyrosine kinases, including PDGFR [33,34], Trk [35,36], insulin-like growth factor receptor [37,38], vascular endothelial growth factor receptors [39,40], and fibroblast growth factor receptors [41]. Here, we focus on EGFR as it is mostly studied.

**Figure 1:**
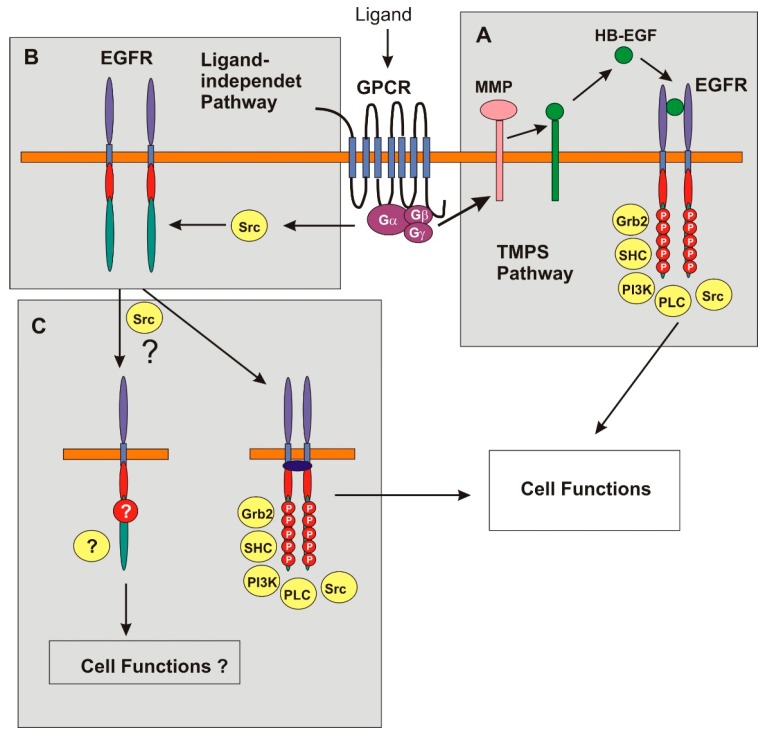
Schematic illustration of EGFR transactivation by GPCR. Two mechanisms have been proposed and supported by the available data: TMPS and ligand-independent intracellular pathways. (**A**) TMPS: In this model, GPCR-induced EGFR transactivation depends on the activation of MMPs that are able to cleave EGFR ligands such as HB-EGF and stimulate ligand shedding. The ligands released into the extracellular space then bind to EGFR and stimulate the dimerization and activation of the receptors, which leads to the activation of signaling cascades; (**B**) Ligand-independent pathway: This pathway involves the activation of intracellular protein tyrosine kinases (PTKs) such as Src family proteins. Src phosphorylates the tyrosines residues in the cytosolic domain of EGFR. The phosphorylated EGFR is able to interact with downstream signaling proteins, leading to the activation of various signaling pathways. Src could be activated by GPCR through different mechanisms; (**C**) Future research: Future research should focus on the EGFR transactivation through ligand-independent Src pathway. It is important to determine how Src activates EGFR, the activation status of Src-activated EGFR, and the effects of Src-activated EGFR on the downstream signaling cascades and cell functions. For example, does Src stimulates the dimerization of EGFR? Does Src phosphorylate all the major phosphor tyrosine (Y) residues of EGFR? What are the downstream signaling pathways activated and what is the physiological consequence of this activation?

Accumulated evidence suggests that there are several mechanisms for the transactivation of the EGFR by GPCR (Figure 1) [6,8,42]. These mechanisms could be classified into two major ones. The first mechanism is the “triple membrane passing signal” (TMPS) pathway (Figure 1A). According to this model, EGFR transactivation by GPCR is controlled by the activation of membrane-bound matrix metalloproteases (MMPs). The most implicated group of MMPs is the ADAM (a disintegrin and metalloprotease) family. MMPs are able to cleave EGFR ligands such as heparin-binding EGF-like factor (HB-EGF), neuregulin, transforming growth factor-α, and amphiregulin. The cleaved ligands are then released into the extracellular space and bind to EGFR, which stimulates the dimerization and activation of EGFR. The activated EGFR is then able to simulate various signaling pathways such as the Ras-Erk pathway and the PI3K-AKT pathway, and regulates various cell functions (Figure 1A). In this model, the signal generated by a GPCR agonist will cross the plasma membrane three times. EGFR transactivation through TMPS pathway has been reported in many cell types following activation by various agonists such as bombesin, 5-hydroxytryptamine, carbachole, angiotensin II, bradykinin, lysophosphatidic acid, endothelin 1, gonadotropin-releasing hormone, phenylephrine, leptin, thrombin, deoxycholic acid, and prostaglandin E2 [6,8,42,43,44]. EGFR transactivation through this mechanism has been implicated in the regulation of many normal cell functions and the growth, development and progression of many diseases such as cancers, kidney disease, and cardiovascular disease.

Under other situations, EGFR is transactivated by GPCR without detectable EGF-like ligands, which suggests that EGFR transactivation by GPCR can also be through intracellular signaling pathways that are ligand-independent (Figure 1B). All of the ligand-independent mechanisms involves the activation of intracellular protein tyrosine kinases (PTKs) such as Src family proteins. The increased PTK activity mediates the phosphorylation of EGFR in its cytosolic domain. The phosphorylated EGFR, in turn, associates with various signaling proteins and initiates the activation of multiple signaling pathways (Figure 1B,C). Several mechanisms have been suggested regarding the pathways leading to the enhanced activity of PTKs [6,8,42,43,45].

A major mechanism requires the ROS production. In this model, activation of GPCRs by agonist stimulates the phosphorylation of p47phox and the activation of NADPH oxidase, which produces reactive oxygen species (ROS) through O_2_ by using NADPH as electron donor. ROS may unbalance the equilibrium of the intracellular phosphorylation and enhance the activity of intracellular PTKs through two mechanisms: (1) inactivation of protein tyrosine phosphatases (PTPs) by oxidation of its cysteine residue in its catalytic site. This leads to the enhanced activation of PTKs; and (2) stimulation of the proteolysis of regulatory proteins that block PTK activity, which also leads to the higher activity of PTKs [6,8,42]. The increased PKT activity stimulates EGFR transactivation.

The ligand-independent intracellular mechanisms without ROS production have also been suggested. Src family kinases have been shown to be associated with GPCRs. The association may be through the direct interaction between Src SH3 domain and the GPCR cytoplasmic domain that contains consensus proline-rich motifs in its C-terminal tail or third intracellular loop. The interaction could also be through the binding to GPCR-associated proteins including the G protein subunits and β-arrestins. This interaction activates Src family kinases, which phosphorylates EGFR at its intracellular domain [6,8,42].

## 5. Progress in the Last Five Years

The research in the field of EGFR transactivation by GPCR is continuously flourish with more than 100 papers published in the last five years. However, most of these papers were reporting the identification of EGFR transactivation by a GPCR, which was not reported before, identification of additional downstream signaling pathways activated by EGFR transactivation, or additional physiological role of EGFR transactivation by GPCR. For example, the GPCRs newly identified to transactivate EGFR or other RTKs include formyl peptide receptor-like (FPRL1) [46], BK B2 receptor (B2R) [47,48], bombesin receptor subtype-3 (BRS-3) [49], the orphan GPCR 101 (GPR101) [50], GPR30/GPER-1 [51,52,53,54], GPR109A [55], GPR48 [56,57], GPR87 [58], urotensin-II receptor [59,60], and all subtypes of α_1_-adrenoceptors (α_1_-AR), including α_1A_-AR, α_1B_-AR, and α_1D_-AR [61].

There are also significant publications that support the role of EGFR transactivation in various diseases including lung cancer [46,49,62,63,64], breast cancer [51,65,66], oral cancer [67], gastrointestinal carcinoma [68,69], Osteoblasts [70], ovarian cancer [52,71,72], hepatocarcinoma [73], head and neck cancer [74], glioblastoma [75], heart disease [55,59], and renal fibrosis [76].

However, there are some developments worth discussion here.

It was reported that angiotensin-(1–7) inhibits EGFR transactivation via a Mas receptor/Src-dependent pathway [77]. In cultured vascular smooth muscle cells, Ang II and glucose induce the transactivation of EGFR, however, this transactivation is attenuated by angitensin-(1–7) via the activation of Mas receptor [77]. They later showed that Ang-(1–7) acts as a pan-ErbB inhibitor via its Mas receptor [78]. It is well established that GPCR transactivates EGFR through Src, this is the first report that GPCR/Src pathway is involved in the inhibition of EGFR transactivation by other GPCRs.

It was reported that GPRC5A directly binds to EGFR, which negatively regulates EGFR activity [79]. Knockout of GPRC5A enhances EGFR-STAT3 signaling in mouse tracheal epithelial cells and GPRC5A expression inhibits EGF-induced EGFR-STAT3 signaling. Furthermore, GPRC5A associates with EGFR via its 7-transmembrane domains, which is required for the inhibition of EGFR. It was further shown that although the 7-transmembrane domain was found to be required for physical interaction and inhibition of EGFR, none of the individual transmembrane domains was critical for inhibition of EGFR signaling. The authors propose that the interaction between GPRC5A and EGFR impedes the dimerization process of EGFR and restrain EGFR from overactivation following ligand binding; however, in the absence of GPRC5A, the negative regulatory loop on EGFR signaling was disrupted, which leads to the persistent activation of EGFR-STAT3 signaling [79]. It was also shown by FRET that kisspeptin-10 directly associated with EGFR, which leads to the transactivation of EGFR [65]. Previously, very little is known regarding the direct association between GPCR and EGFR. It was reported that somatostatin receptors SSTR1 and SSTR5 associate with EGFR in the absence of agonist, however, SSTR1 and SSTR5 disassociate from EGFR following agonist stimulation. This disassociation modulates EGFR activation and the downstream signaling pathways [80].

GPCR may regulate EGFR activity through the regulation of EGFR degradation pathway. It has been shown that E2/S1P stimulates Cdc42 and blocks EGFR degradation. However, Cdc42 knockdown restores rapid EGFR degradation following E2/S1P activation. Moreover, activation of Cdc42 by E2 is prevented by inhibiting S1P3 receptors, which suggests that S1P receptor plays important role in E2 signaling. This finding provides a novel mechanism to further define the role of E2/S1P on the transactivation of EGFR in breast cancer cells [66]. It was also reported that disruption of lipid raft blocked TGR5 and EGFR interaction. TGR5 signals from plasma membrane rafts that facilitate EGFR interaction and transactivation [81].

It was reported that activation of P2Y_2_R in human salivary gland cells stimulates the release of neuregulin through metalloprotease, which results the activation of both EGFR and ErbB3 by promoting the heterodimerization of EGFR and ErbB3 [82]. A recent study also support the role of focal adhesion kinase (FAK) in the transactivation of EGFR by GPCR. It was found that FAK coordinates the dynamic assembly and disassembly of a protein complex including both GABA receptor and its effectors, which is essential for the transactivation of EGFR [83]. Moreover, a functional siRNA screen identifies a suite of genes encoding several proteins involved in EGFR transactivation by the angiotensin type 1 receptor (AT_1_R). These proteins include TRIO, BMjX and CHKA [84].

Some new technologies have also been used to study the transactivation of EGFR and other RTKs by GPCR. Besides the abovementioned FRET technology [65] and siRNA [84], spatial intensity distribution analysis (SpIDA) was also used [85]. SpIDA is based on image analysis, which is able to directly examine the trafficking and oligomerization of endogenous proteins within a single cell. Using this method, it was observed that transactivation of EGFR and TrkB occurred on the same timescale and was directly limited by GPCR activation but the transactivation is independent of the type of the GPCR [85].

## 6. Challenges and Future Research

As discussed above, while the transactivation of EGFR or other RTK by GPCR continues to attract significant interest from the research community, few significant progress has been made during the last five years. In order to move this research field further, future research has to identify and overcome the various challenges in this field. We believe that the biggest challenge is to understand the molecular mechanism underlying the transactivation of EGFR through ligand-independent pathways, demonstrate the role of transactivation in various disease and develop therapies to target GPCR-mediated EGFR transactivation.

EGFR transactivation by HB-EGF through TMPS mechanism is well defined. EGFR activation by released HB-EGF through TMPS should be quite similar to that by direct HB-EGF stimulation. The mechanism of EGFR activation by ligand is well defined. The activation status and the effects of activated EGFR on downstream signaling cascades and cellular functions are well established. Indeed, the available data regarding EGFR transactivation by cleaved HB-EGF induced by GPCR activation support that the cleaved HB-EGF stimulates the dimerization and phosphorylation of EGFR, which activates various intracellular signaling pathways in the same way as EGFR activated directly by HB-EGF. For example, transactivation of EGFR by released HB-EGF through LPA receptor activation by LPA in Cos-7 cells induces the activation of major EGFR signaling pathways including ERK, PLC-γ1 and PI3K-Akt pathways, which is similar to the direct activation of EGFR by HB-EGF [42].

The weak area that needs specific attention and further research is the transactivation of EGFR by GPCR through ligand-independent pathways (Figure 1C). Regardless how Src is activated by GPCR, it is important to determine how Src activates EGFR, the activation status of Src-activated EGFR, and the effects of Src-activated EGFR on the downstream signaling cascades and cell functions. For example, does Src stimulates the dimerization of EGFR? If yes, how? If not, does Src phosphorylate all the major phosphor tyrosine (Y) residues including Y992, Y1045, Y1068, Y1086, Y1148, and Y1173? If Src only selectively phosphorylates some tyrosine residues of EGFR, what downstream signaling pathways will be active by this partially activated EGFR? What is the physiological consequence of this activation? It was pointed out in a previous review that ligand-independent EGFR transactivation may cause the incomplete activation of EGFR and the downstream signaling cascades [42]. However, no progress has been made.

The only well-established Src-phosphorylated tyrosine residue in EGFR is Y845 [86,87]. However, the effects of Y845 phosphorylation on EGFR activation, the activation of downstream signaling cascade, and the induced-cellular responses are not well defined [86]. For example, Y845 phosphorylation have been shown to regulate cell apoptosis, but some researches indicate an anti-apoptotic role for Y845 phosphorylation [88,89,90,91] and others indicates a pro-apoptotic role of Y845 phosphorylation [92,93,94]. A previous study indicates that EGFR Y845 phosphorylation by Src has no effect on the autophosphorylation of EGFR [87]. However, different from this report, another study shows that EGFR-Y845F mutant augments ligand-stimulated EGFR phosphorylation on its C-terminal tyrosine residues and enhances ligand-induced DNA synthesis [95]. It was further shown that EGFR transphosphorylation at Y845 positively regulates its autophosphorylation, kinase activity and effects on cell proliferation [96].

If only Y845 is phosphorylated in transactivated EGFR through Src by GPCR and all of the major phosphor tyrosine residues are phosphorylated in EGFR transactivated through HB-EGF by GPCR, the overall effects on cellular response will greatly different. Thus, it is essential to understand the mechanisms underlying the transactivation when studying GPCR-induced transactivation of EGFR.

Another research focus should be the clinical relevance of EGFR transactivation by GPCR. While many studies have suggest the relevance of EGFR transactivation by GPCR to various diseases, there is very few clinical data to support the relevance. Most data supporting the cancer-relevance demonstrate the existence of transactivation of EGFR in various cancer cell lines in the lab. Some data are generated with xenograft in mice. Data obtained under these experimental setting show that both EGFR and certain GPCR are co-overexpressed and simultaneous inhibition of both EGFR and GPCR leads to additive or synergistic growth inhibition in cancers [5,62,74,75]. However, as GPCRs have also been shown to stimulate cancer cell proliferation in an-EGFR independent pathway [5,97,98,99], the observed synergism cannot be convincingly attribute to the role of EGFR transactivation by GPCR.

Very few data were obtained with patient samples. It was recently reported that glioblastoma specimens from patients have a 4–17-fold increase in dopamine receptor D2 (DRD2) mRNA or 2–4-fold enhancement in protein expression [75]. They also show that the anti-glioblastoma activity of dopamine antagonists is synergistic when combined with EGFR inhibition in cultured cancer cells and in xenograft-bearing mice [75].

To demonstrate the disease-relevance of EGFR transactivation, the most important future research is to obtain data from patients. While it is a difficult task distinguish the effects of EGFR and GPCR activation from the effects of EGFR transactivation by GPCR in a clinical setting, it is very feasible to at least collect significant data to determine whether there is the co-overexpression of EGFR and GPCR in patients and whether this co-overexpression correlates with poor prognosis. It is also important to show whether the combined inhibition of EGFR and GPCR confers synergism in patients by using clinically approved agents. If EGFR transactivation by GPCR plays important role beyond the activation of EGFR and GPCR in diseases, specific disruption of the cross-talk between EGFR and GPCR without inhibition of EGFR and GPCR should have significant inhibitory effects on disease progress. Thus, identification the agents that specifically and efficiently disrupt the transactivation of EGFR by GPCR is an important task in the future research. A likely target of these agents is the ADAM family of matrix metalloproteases if the transactivation of EGFR is through TMPS pathway. If the transactivation of EGFR is through a ligand-independent pathway, the likely target is Src family protein tyrosine kinases. While Src plays important role in cancer independent its role in EGFR transactivation and has been targeted for cancer therapy [100,101], the identification of its role in EGFR transactivation may provide an additional reason to target Src for cancer therapy.

## 7. Conclusions

Since the first report of EGFR transactivation of GPCR, this topic has attracted enormous attention from the research community. Significant progress has been made, including the elucidation of the mechanisms underlying the transactivation. More and more GPCRs have been identified to be able to transactivate EGFR. Physiological and disease relevance of EGFR transactivation by GPCR have been supported by more data. However, despite continued interest in the research community, no significant progress has been made during the last five years. To significantly advance this research field, future research should be focused on both the molecular mechanisms regarding the transactivation of EGFR through ligand-independent and Src-dependent pathways. The most important task in the future research is to demonstrate the disease relevance of EGFR transactivation by GPCR in clinical settings, and identify potential therapeutic agents to specifically target EGFR transactivation by GPCR.
